# The Right Time for a Synapse to Change: Windows and Mechanisms of Multiday Training Trials

**DOI:** 10.1523/JNEUROSCI.1981-25.2026

**Published:** 2026-03-30

**Authors:** Rong-Yu Liu, Yili Zhang, Roberta Calvo, Paul Smolen, John H. Byrne

**Affiliations:** ^1^Department of Neurobiology and Anatomy, W.M. Keck Center for the Neurobiology of Learning and Memory, McGovern Medical School at the University of Texas Health Science Center at Houston, Houston, Texas 77030; ^2^Departments of Electrical and Computer Engineering and Psychological Sciences, Rice University, Houston, Texas 77005

**Keywords:** computational modeling, kinase, long-term synaptic plasticity, neuronal excitability, spaced learning, transcription factor

## Abstract

Although learning over multiple days is more effective than a single day of training, the underlying cellular mechanisms of repeated training trials remain poorly understood. With a combination of empirical and computational approaches, we determined a critical time window for a second stimulus block of a multiday training protocol to augment long-term synaptic facilitation (LTF) of the *Aplysia* sensorimotor synapse and long-term enhancement of neuronal excitability (LTEE), two cellular correlates of learning and memory. A second stimulus block delivered 24 h after the first block significantly enhanced LTF and LTEE, but was without effect at 18 or 32 h. This spacing effect appears due, at least in part, to the dynamics of competition between the transcription activator cAMP response element-binding protein 1 (CREB1) and repressor CREB2. The timer mechanism is intrinsic to individual neurons, as LTEE exhibited this critical temporal window in isolated sensory neurons. These findings suggest the dynamics of transcription factors function as a cellular timer that establishes a window of eligibility for a second learning trial to enhance memory.

## Significance Statement

A dogma in the field of learning and memory, and the science of education, is that learning over multiple days with an approximate 24 h interval is more effective than a single day of training. Little is known about the consequences of retarding or advancing that interval or about the neuronal mechanisms underlying the effectiveness of multiday learning. Using cellular analogs of learning, this study shows that the 24 h interval is fortuitous; 18 or 32 h intervals are significantly less effective. A molecular hour-glass-like timer mechanism involving the transcription factors CREB1 and CREB2 appears to generate a critical learning window. The study reveals a simple biological timer underlying multiday learning efficacy, with broad implications for neuroscience and education.

## Introduction

Numerous studies of learning and memory use training protocols that repeat trials over multiple days, typically with 24-h inter-training intervals (Table 1; Pinsker et al., 1973; Frost et al., 1985; Wainwright et al., 2002; Cepeda et al., 2006; Goedert and Miller, 2008; Hu et al., 2011; Dobson, 2012; Smolen et al., 2016; Smith and Scarf, 2017; Verhoeven and Newell, 2018; Correa et al., 2022; Mastrorilli et al., 2022). However, little is known about the consequences of advancing or delaying the 24 h interval, or about the molecular mechanisms underlying multiday learning. To address these questions, we exploited the technical advantages of the *Aplysia* sensorimotor synapse and isolated sensory neuron (SN) cultures to investigate molecular mechanisms that determine effective interblock intervals (IBIs) for a multiday training protocol. This protocol elicits long-term synaptic facilitation (LTF) in sensory-motor neuron cocultures, a cellular correlate of long-term sensitization, a form of long-term memory (LTM; Frost et al., 1985; Cleary et al., 1998). A single-block “Standard” protocol consisting of five evenly spaced pulses of serotonin (5-HT) induces LTF that persists for at least 24 h (Montarolo et al., 1986; Martin et al., 1997; Chain et al., 1999), but decays substantially by 48 h (Zhang et al., 2011; Liu et al., 2017). In contrast, repeated stimulus blocks with a fixed 24 h IBI lead to LTM and LTF that persist for 6–7 d (Pinsker et al., 1973; Frost et al., 1985; Wainwright et al., 2002; Hu et al., 2011), suggesting some “trace” is left after the first stimulus block that may facilitate the effects of the next.

Training protocols activate kinases, gene transcription, and protein translation (Kandel, 2001; Alberini, 2009; Campbell and Wood, 2019), and these changes persist for hours to days across animal species (Alberini, 2009; Byrne and Hawkins, 2015; Smolen et al., 2016, 2019; Campbell and Wood, 2019; Rivi et al., 2020; Rahmani and Chew, 2021; Roselli et al., 2021). In *Aplysia*, complex dynamic changes in activation of multiple kinases [e.g., the mitogen-activated protein kinase (MAPK) isoforms extracellular signal-regulated kinase (ERK) and p38 MAPK, ribosomal S6 kinase (RSK), protein kinase A (PKA)] occur over 24 h following 5-HT treatment (Muller and Carew, 1998; Liu et al., 2020; Zhang et al., 2023, 2025). The 18 h time point is particularly interesting because, at this time, all four learning-related kinases are activated. In contrast, by 24 h the kinase activities return towards basal levels (Zhang et al., 2023, 2025). RSK and PKA contribute to activation of the transcription activator CREB1 and promote LTF (Bartsch et al., 1998; Liu et al., 2020), whereas p38 MAPK activates the transcription repressor CREB2 and inhibits LTF (Guan et al., 2003).

Based on these findings, we predicted that CREB1 and CREB2 activation would exhibit rich dynamics, and that competition between these opposing transcription factors would generate temporal “windows” such that a second training trial might be more or less effective, depending on its timing. To test this prediction, we examined the dynamics of activation of CREB1 and CREB2 at 18, 24, and 32 h after an initial stimulus block. The 18 h time point was selected because, at that time, kinases, CREB1, and CREB2 were all active, generating competition between activation and repression of transcription. The 24 h time point was selected based on its known efficacy in multiblock training in many species (Table 1). At that time, we hypothesized that the competition between CREB1 and CREB2 would favor CREB1, allowing a second stimulus to add to residual CREB1 activity to enhance LTF. The 32 h time point was chosen based on a computational model describing competition between a transcription activator and a repressor (Fig. S2).

**Table 1.** Examples of long-term synaptic plasticity and learning and memory induced by multiday training

Study	Interval	References
Aplysia long-term sensitization	1 d	Pinsker et al. (1973)
Aplysia long-term sensitization	1 d	Frost et al. (1985)
Aplysia long-term sensitization	1 d	Wainwright et al. (2002)
Aplysia LTF	1 d	Hu et al. (2011)
Human visuomotor skill	1 d	Goedert and Miller (2008)
Human auditory perceptual learning	1 d	Molloy et al. (2012)
Human endoscopic skill	1 d	De Win et al. (2013)
Human vocabulary learning and visual acuity performance	7 min–24 h	Kornmeier et al. (2014)
Human motor learning and performance	1 d	Verhoeven and Newell (2018)
Mouse spatial memory	1 d	Mastrorilli et al. (2022)

Electrophysiological approaches were used to examine whether these transcription factor dynamics correlated with a critical time window for the second stimulus block of a multiday training protocol to effectively augment LTF as well as a second correlate of long-term memory, long-term enhancement of excitability (LTEE) in SN cultures.

## Materials and Methods

### Neuronal cultures and treatments

*Aplysia* are simultaneous hermaphrodites, meaning each animal functions as both male and female. Isolated sensory neurons (SNs) or SN-L7 motor neuron (MN) cocultures from *Aplysia californica* [National Aplysia Resource (NAR), University of Miami] were prepared according to conventional procedures (Liu et al., 2008, 2011a,b, 2020; Zhang et al., 2011). Dishes of isolated SN cultures were plated with 10–12 SNs. Dishes of SN-MN cocultures were plated with a single SN and a single L7 MN. Cultures were maintained in growth medium (50% adjusted L15 with 1.2 mg/ml of ʟ-glutamine and 50% hemolymph) in relatively constant darkness at 18°C in a Thermo Scientific Forma 3900 incubator. The incubator was equipped with a light shield, but some light (0.5–4 lux) entered during weekdays. Cultures were exposed to ambient room light (400–650 lux) only during medium changes (minutes), 5-HT treatment (85 min for the first stimulus block and 5 min for the second stimulus block), and the electrophysiology recording (1–2 h for pretest and posttest). The first stimulus block was administered in the afternoon, typically between 2:00 and 4:00 P.M. The second stimulus block was delivered on the following day at various times, depending on the IBI. Application times were between 8:00–10:00 A.M. for IBI of 18 h, 2:00–4:00 P.M. for IBI of 24 h, and 8:00–10:00 P.M. for IBI of 32 h. The pretests for LTF and LTEE were performed immediately before the first stimulus block and usually started between 8:00 and 10:00 A.M. The 2 and 3 d posttests were performed between 2:00 and 4:00 P.M.

SB203580 (SB; Millipore Sigma, catalog #559389) was used to block p38 MAPK activity. A concentration of 3 µM was used based on its effectiveness in prior studies in *Aplysia* (Guan et al., 2003; Liu et al., 2014; Zhang et al., 2017)*.* Four dishes of SNs from the same animal were used. Each dish was given a different treatment: Vehicle (Veh), 5-HT (S) alone, SB alone, or S + SB. SNs were fixed at 18 h followed by pCREB2 or pCREB1 immunostaining or fixed 24 h after SB and a second stimulus block treatment. The same procedures were used with SN-MN cocultures to examine whether blocking the effects of late p38 MAPK activation enhances LTF induced by a second stimulus block.

### Immunofluorescence studies in isolated sensory neuron cultures

*Aplysia* SN cultures were maintained in growth medium for 5–6 d, and at least 2 h before the 5-HT treatments, the growth medium was replaced with 50% L15 and 50% artificial seawater (ASW; L15:ASW, 1:1 v/v). SNs were then treated with five 5 min pulses of either vehicle or 50 µM 5-HT (diluted in L15:ASW) with an interpulse interval of 20 min.

ERK and p38 MAPK drive the activation of CREB1 and CREB2, respectively, and the dynamics of ERK activity after three LTF-inducing protocols have been characterized recently (Zhang et al., 2023). In this study, we compared the phosphorylation of CREB1 and CREB2 at 18, 24, or 32 h after the standard protocol. The antibody to phosphorylated CREB1 (pCREB1, Genemed Synthesis) utilized the Ser85-phosphorylated CREB1 peptide KKRREILTRRPSYRK epitope (Mohamed et al., 2005). Anti-pCREB2 antibody utilized a phosphorylated CREB2 hybrid peptide epitope (SPPDSPEQGPSSPET) constructed to juxtapose the sequences immediately surrounding two putative phosphorylation sites (Genemed; Mohamed et al., 2005) with phosphorylation mediated by p38 MAPK (Liu et al., 2014).

Immunofluorescence procedures followed those of Liu et al. (2011a). Briefly, cells were fixed at 18, 24, or 32 h after first stimulus block in an ice-cold solution of 4% paraformaldehyde in phosphate-buffered saline (PBS) containing 30% sucrose for 30 min at room temperature. After three rinses in PBS, fixed cells were blocked for 30 min at room temperature in Superblock blocking buffer (Thermo Fisher, catalog #37515)/0.2% Triton X-100/3% normal goat serum and subsequently incubated overnight at 4°C with pCREB1 antibody (1:250 dilution) or pCREB2 antibody (1:250 dilution) in antibody dilution buffer (Superblock blocking buffer/0.1% Triton X-100/3% normal goat serum). Goat anti-rabbit secondary antibody conjugated to Cy-3 (Jackson ImmunoResearch Lab, catalog #NC9771594 1:100 dilution) was applied in the same antibody dilution solution for 1 h at room temperature. After two PBS washes, nuclei were stained with DAPI (Sigma-Aldrich, catalog #D9542, 2 µg/ml) for 15 min in the dark at room temperature. Finally, cells were rinsed twice with PBS and mounted to slides. Images were obtained with a Zeiss LSM 800 confocal microscope using a 63× oil immersion lens. A *z*-series of optical sections through the cell body (0.5 µm increments) were taken, and the section through the middle of the nucleus was used for analysis of mean fluorescence intensity with ImageJ (version 1.54k, National Institutes of Health; Liu et al., 2020; Zhang et al., 2021). Five to ten neurons on each coverslip were analyzed, and measurements from neurons on the same coverslip were averaged. At each time point two dishes of SNs from the same animal were used: one was treated with 5 pulses of 5-HT, the other was treated with vehicle as control. Because immunoreactivity of pCREB1 and pCREB2 was predominantly located in the nucleus, we focused on changes in the nucleus. Data were analyzed with a paired *t* test. All experiments were performed in a blind manner so that the investigator analyzing the images was unaware of the treatment. The number of samples (*n*) reported indicates the number of SN culture dishes assessed.

To examine the possibility that transduction mechanisms in cultured SNs were regulated by a diurnal modulation, SNs were changed to L15:ASW for 2 h and then treated with a single 5 min pulse of 5-HT, delivered at 8:00 A.M., 2:00 P.M., or 10:00 P.M. After the 5 min 5-HT treatment, SNs were kept in L15:ASW and returned to the incubator for 40 min. SNs were then fixed for immunostaining of pERK using an anti-pERK antibody (Cell Signaling, catalog #4370, 1:400 dilution), following the same immunofluorescence and confocal imaging procedures described above.

### Electrophysiology

**Figure 1.**
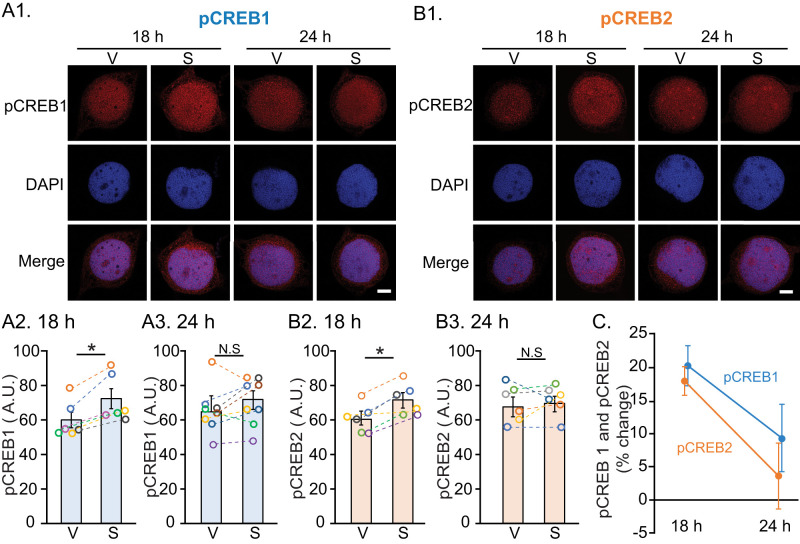
Phosphorylation of CREB1 and CREB2 after the first stimulus block. ***A1***, Confocal images of SNs after pCREB1 immunostaining. DAPI was used as a nuclear counterstain. Merged images indicated immunoreactivity to pCREB1 was highest in the nucleus. ***A2***, Summary data. Different colored circles in each group indicate individual experiments, and the same color in different groups represents one set of experiments. Data in this figure and all other figures are represented as mean ± SEM. ∗*p* < 0.05. ***A3***, No significant (N.S) difference was found in pCREB1 between 5-HT (S) and vehicle (V) groups. ***B1***, Confocal images of SNs after pCREB2 immunostaining. Immunoreactivity to pCREB2 was also highest in the nucleus. ***B2***, ***B3***, Summary data. ***C***, Changes in pCREB1 and pCREB2 at 18 and 24 h. Scale bars, 15 µm.

**Figure 2.**
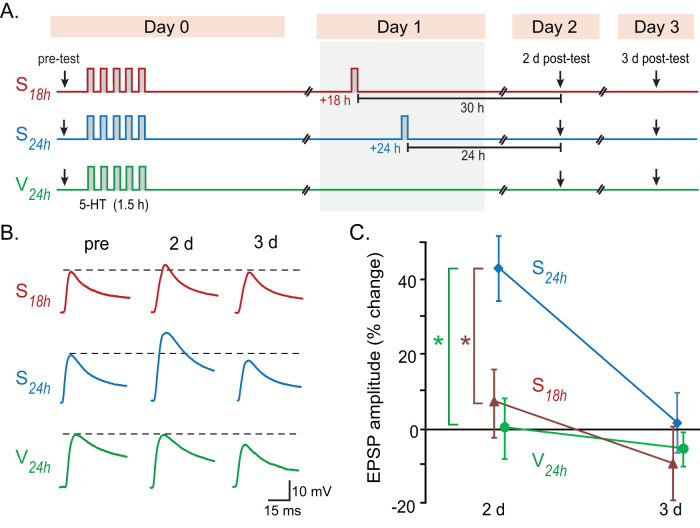
LTF induced by two-block stimuli with varying IBIs. ***A***, Protocols for two-block 5-HT treatment with different IBIs. ***B***, Representative excitatory postsynaptic potential (EPSP) recordings. ***C***, Summary data showing that LTF was prolonged by delivering a second block at 24 h, but not 18 h.

**Figure 3.**
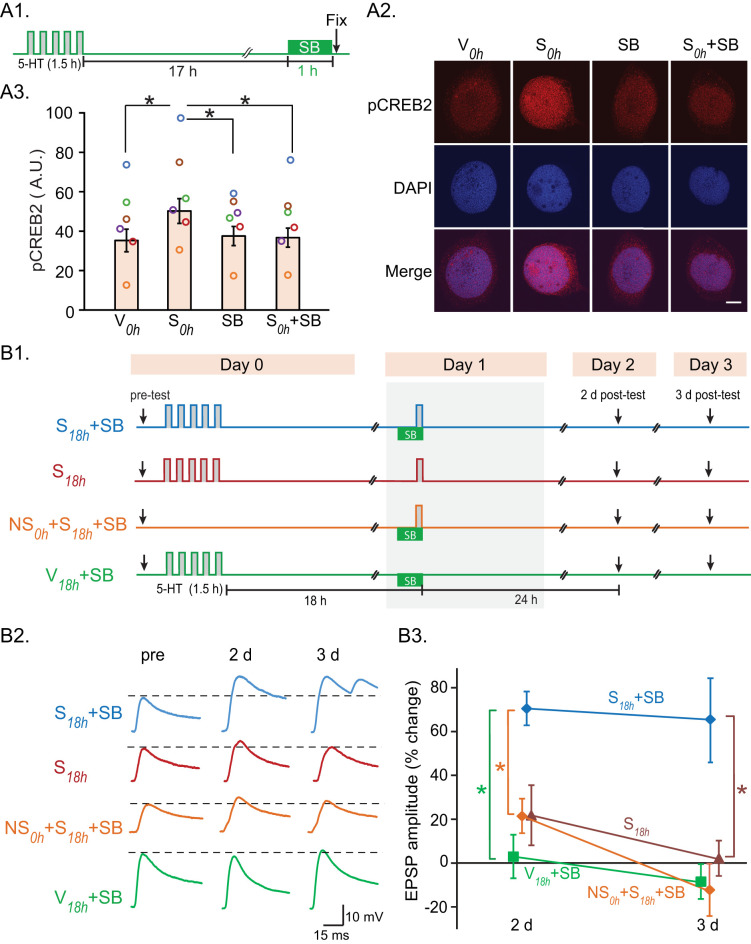
LTF was prolonged by inhibiting the 5-HT-induced increase in phosphorylation of CREB2 at 18 h. ***A***, The pCREB2 increase was attenuated by the p38 MAPK inhibitor SB203580 (SB). ***A1***, Protocol. SB (3 µM) was applied 17 h after the first stimulus block, in the absence of a second stimulus block. SNs were fixed immediately after 1 h of SB treatment and processed for immunostaining. ***A2***, Confocal images after immunostaining. Scale bars, 15 µm. ***A3***, Summary data. ***B***, LTF was prolonged by inhibition of p38 MAPK. ***B1***, Protocol for SB application and electrophysiological testing for LTF. ***B2***, Representative EPSPs. ***B3***, Summary data.

For LTF experiments, SN-MN cocultures were prepared as described previously (Angers et al., 2002) and maintained in an 18°C incubator with growth medium for 4 d to allow the synapse to form and stabilize. On the 5th day in culture, cocultures were changed to L15:ASW before electrical recording. An initial EPSP was triggered to confirm a synaptic connection. A blunt patch stimulating glass electrode filled with modified L15:ASW was used to extracellularly stimulate the presynaptic SN, and EPSPs were recorded from the MN with 10–20 MΩ sharp electrodes filled with 3 M potassium acetate. MNs with resting membrane potential more positive than −40 mV or input resistance smaller than 10 MΩ were excluded for further analysis. Neurons used in the analysis had a membrane potential in the range of −40 to 65 mV and input resistance in the range of 10–40 MΩ. Data acquisition was performed using pClamp (Clampex 10.7, Molecular Devices). To examine LTF, the standard 5-HT protocol was used as the first stimulus block of 2 d training. This standard protocol consists of five pulses of 5 min applications of 5-HT (50 µM) with 20 min interpulse intervals. The second stimulus block, consisting of one 5 min pulse of 5-HT, was delivered at 18, 24, or 32 h after the end of the first block. After 5-HT treatment, cocultures were changed to culture medium and placed back to the incubator. For statistical analysis of LTF, the amplitude of the EPSP was measured before (pretest), 48 h (2 d post), and 72 h (3 d post) for Figures 1–3 after exposure to 5-HT or vehicle (posttest). Synaptic facilitation was defined as the percent change in EPSP amplitude from pretest.

For LTEE, separate groups of isolated SNs were used. SN cultures were prepared as previously described (Liu et al., 2011a) and maintained in an 18°C incubator for 5 d. On Day 6, neurons were impaled with a single microelectrode (10–20 MΩ resistance) filled with 3 M potassium acetate and current clamped at −45 mV. Input resistance was measured by applying 0.1 nA of hyperpolarizing current for 2 s. Firing threshold was measured by applying 2 s of depolarizing current in increasing increments of 0.1 nA until an action potential (AP) was triggered. The lowest current intensity necessary to fire a single AP was considered the firing threshold. Excitability was measured by counting the number of APs triggered by applying 0.5 nA of depolarizing current for 2 s. When the firing threshold of SNs was equal to or above 0.5 nA, but less than 1.0 nA, 1.0 nA of depolarizing current was used to measure excitability. The firing threshold for SNs is typically 0.2–0.7 nA. SNs were excluded from further use if cells had resting potentials more positive than −35 mV or failed to respond to depolarizing current up to 1.0 nA. Data acquisition was performed using pClamp (Clampex 11.3, Molecular Devices). To examine LTEE, the same 2 d training protocols described for LTF were used. SN cultures were maintained in culture medium, except during 5-HT treatment and LTEE recordings. Two to five neurons in each dish were recorded and measurements from SNs in the same dish were averaged. For statistical analysis of LTEE, the number of APs was measured before (pretest), 48 h (2 d post), and 72 h (3 d post) after the first stimulus block of 5-HT. LTEE was defined as the percentage change in APs from pretest.

### Model development

See Supplemental Information, Figures S1, S2, and Table S1.

## Results

### Phosphorylation of CREB1 and CREB2 at 18 and 24 h after LTF induction

**Figure 4.**
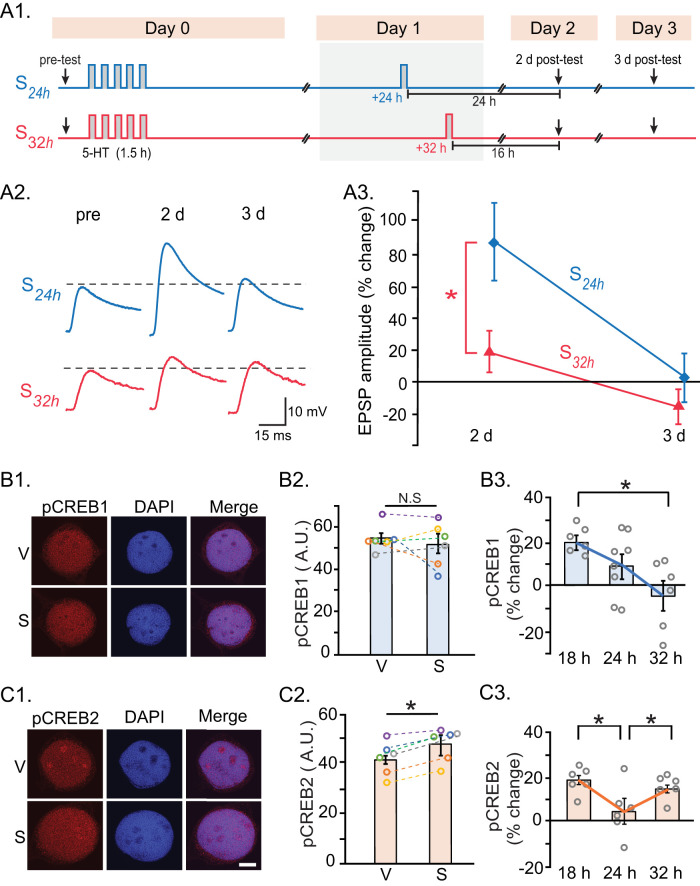
Changes in LTF induced by a two-block protocol with IBI of 32 h and changes in pCREB1 and pCREB2 after initial stimulus block. ***A***, An IBI of 32 h resulted in significantly less LTF, compared with an IBI of 24 h. ***A1***, Protocols for two-block stimulations. ***A2***, Representative EPSPs. ***A3***, Summary data. ***B***, Changes in pCREB1 32 h after the initial block of five pulses of 5-HT, in the absence of a second block. ***B1***, Confocal images of SNs after pCREB1 immunostaining 32 h after 5-HT. ***B2***, Summary data for the immunoreactivity of pCREB1 at 32 h. ***B3***, Summary data for the changes of pCREB1 at 18, 24, and 32 h after 5-HT. Data for 18 and 24 h are replotted from Figure 1*C*. ***C***, Changes in pCREB2 after 5-HT. ***C1***, Confocal images of SNs after pCREB2 immunostaining at 32 h. ***C2***, Summary data for the immunoreactivity of pCREB2 at 32 h. ***C3***, Summary data for the changes of pCREB2 at 18, 24, and 32 h after 5-HT. Scale bars, 15 µm.

**Figure 5.**
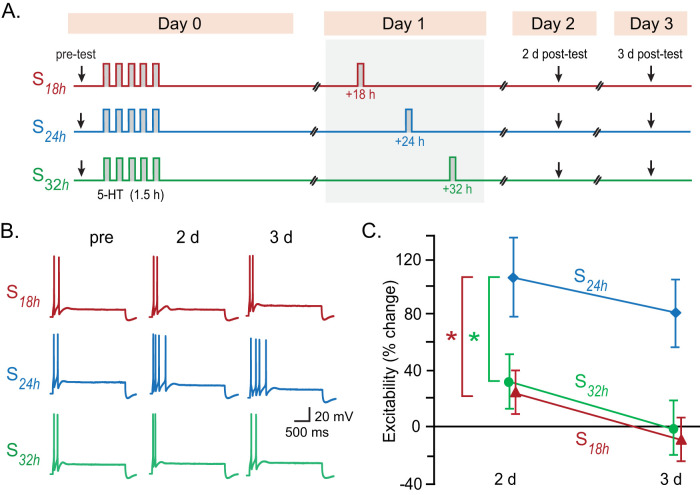
LTEE induced by a two-block protocol with varying IBI. ***A***, The second stimulus was delivered at 18, 24, or 32 h after the first. ***B***, Representative intracellular recordings from isolated sensory neurons recorded before, and 2 and 3 d after, the two-block treatment. ***C***, Summary data. A second block delivered at 24 h after the first block (*S*_24h_) prolonged LTEE up to 3 d posttreatment, an effect significantly greater than that observed with a second block applied at 18 h (*S*_18h_) or 32 h (*S*_32h_).

As a first step to test the hypothesis that the status of CREB1 and CREB2 impacts the consequences of a second stimulus block delivered the next day, levels of phosphorylated (i.e., activated) CREB1 (pCREB1) and CREB2 (pCREB2) were measured after the standard protocol (five 5 min pulses of 5-HT spaced 20 min apart; Fig. 1). Eighteen hours after 5-HT, nuclear pCREB1 increased significantly compared with the vehicle (V)-treated control (Fig. 1*A2*). At 24 h, pCREB1 still appeared elevated, but the change was not statistically significant (Fig. 1*A3*). Similarly, 5-HT treatment significantly increased pCREB2 at 18 h (Fig. 1*B2*). However, at 24 h, pCREB2 returned to basal levels (Fig. 1*B3*). A two-way ANOVA revealed that for pCREB1 and pCREB2, the decreases from 18 to 24 h were significant (Fig. 1*C*). Statistical analyses for experiments of Figures 1–5 are provided in Table 2.

**Table 2.** Detailed statistics

Figure 1, immunostaining				
Measurements	Statistical test	Sample size (*n*)	Statistics	*p* value
Figure 1*A2*	Paired *t* test	V: 6; S: 6	*t*_(5)_ = 6.207	*p* = 0.002
Figure 1*A3*	Paired *t* test	V: 8; S: 8	*t*_(7)_ = 1.416	*p* = 0.200
Figure 1*B2*	Paired *t* test	V: 6; S: 6	*t*_(5)_ = 7.981	*p* < 0.001
Figure 1*B3*	Paired *t* test	V: 6; S: 6	*t*_(5)_ = 0.511	*p* = 0.631
Figure 1*C*	Two-way ANOVA		Time: *F*_(1,22)_ = 8.79	*p* = 0.007
Figure 2*C*, LTF				
Measurements	Statistical test	Sample size (*n*)	Statistics	*p* value
LTF	Two-way RM ANOVA	*V*_24h_: 6 *S*_18h_: 6 *S*_24h_: 7	Protocols, *F*_(2,16)_ = 4.407	*p* = 0.036
			Time, *F*_(1,16)_ = 15.86	*p* = 0.001
	Student–Newman–Keuls (SNK) pairwise comparison		2 d, *S*_24h_ versus *V*_24h,_ *q *= 5.243	*p* = 0.003
			2 d, *S*_24h_ versus *S*_18h,_ *q* = 4.442	*p* = 0.004
			2 d, *S*_18h_ versus *V*_24h,_ *q* = 0.0595	*p* = 0.677
Pretest EPSP	One-way ANOVA	Same as above	*F*_(2,17)_ = 0.26	*p* = 0.774
Figure 3				
Measurements	Statistical test	Sample size (n)	Statistics	*p* value
Figure 3*A3*	One-way RM ANOVA	*V*_0h:_ 6 *S*_0h:_ 6 SB: 6 *S*_0h_ + SB: 6	*F*_(3,15)_ = 6.701	*p* = 0.004
	Pairwise multiple comparison (SNK)		*S*_0h_ versus *V*_0h,_ *q* = 5.46	*p* = 0.008
			*S*_0h_ versus SB, *q* = 5.09	*p* = 0.007
			*S*_0h_ versus *S*_0h_ + SB, *q* = 4.93	*p* = 0.003
			SB versus *S*_0h_ + SB, *q* = 0.32	*p* = 0.826
			SB versus *V*_0h,_ *q* = 1.096	*p* =0.451
Figure 3*B3*, LTF	Two-way RM ANOVA	*S*_18h_: 5 *S*_18h_ + SB: 6 *V*_18h_ + SB: 6 NS_0h_ + *S*_18h_ + SB: 7	Treatment, *F*_(3,17)_ = 15.34	*p* < 0.001
			Time, *F*_(1,17)_ = 7.27	*p* = 0.015
	Pairwise comparisons (SNK)		Group 2 versus 1_,_ *q* = 6.67	*p* < 0.001
			Group 2 versus 3, *q* = 8.35	*p* < 0.001
			Group 2 versus 4, *q* = 7.36	*p* < 0.001
			Group 1 versus 3, *q* = 1.61	*p* = 0.501
			Group 1 versus 4, *q* = 0.66	*p* = 0.644
			Group 4 versus 3, *q* = 0.95	*p* = 0.509
Pretest EPSP	One-way ANOVA	Same as above	*F*_(3,23)_ = 2.049	*p* = 0.139
Figure 4, LTF induced by 2 blocks with IBIs of 24 and 32 h				
Measurements	Statistical test	Sample size (*n*)	Statistics	*p* value
LTF	Two-way RM ANOVA	*S*_24h_: 5 *S*_32h_: 6	Protocols: *F*_(1,8)_ = 7.787	*p* = 0.017
			Time: *F*_(1,8)_ = 25.534	*p* < 0.001
	Pairwise comparisons (SNK)		*S*_24h_ versus *S*_32h,_ *q* = 3.854	*p* = 0.02
			2 d post versus 3 d post, *q* = 7.268	*p* = 0.001
Pretest EPSP	*t* test	Same as above	*t*_(11)_ = 0.0381	*p* = 0.97
pCREB1 (32 h)	Paired *t* test	V: 6; S: 6	*t*_(5)_ = 0.761	*p* = 0.481
pCREB2 (32 h)	Paired *t* test	V: 6; S: 6	*t*_(5)_ = 6.249	*p* = 0.002
Changes of pCREB1 at 18, 24 and 32 h	One-way ANOVA	*S*_18h_: 6 *S*_24h_: 8 *S*_32h_: 6	*F*_(2,17)_ = 4.988	*p* = 0.02
	Pairwise comparisons (SNK)		*S*_18h_ versus *S*_32h_, *q* = 4.457	*p* = 0.015
			*S*_24h_ versus *S*_32h_, *q* = 2.633	*p* = 0.08
			*S*_18h_ versus *S*_24h_, *q* = 2.132	*p* = 0.150
Changes of pCREB2 at 18, 24 and 32 h	One-way ANOVA	*S*_18h_: 6 *S*_24h_: 6 *S*_32h_: 6	*F*_(2,15)_ = 4.905	*p* = 0.023
	Pairwise comparisons (SNK)		*S*_18h_ versus *S*_24h_, *q* = 4.265	*p* = 0.022
			*S*_18h_ versus *S*_32h_, *q* = 1.097	*p* = 0.45
			*S*_24h_ versus *S*_32h_, *q* = 3.168	*p* = 0.041
Figure 5, LTEE induced by 2 blocks with IBIs of 18, 24 and 32 h				
Measurements	Statistical test	Sample size (*n*)	Statistics	*p* value
LTEE	Two-way RM ANOVA	*S*_18h_: 8 *S*_24h_: 10 *S*_32h_: 8	Protocols: *F*_(2,23)_ = 4.95	*p* = 0.016
			Time: *F*_(1,23)_ = 5.97	*p* = 0.023
	Pairwise comparisons (SNK)		*S*_18h_ versus *S*_24h_, *q* = 3.94	*p* = 0.028
			*S*_24h_ versus *S*_32h_, *q* = 3.60	*p* = 0.018
			*S*_18h_ versus *S*_32h_, *q* = 0.31	*p* = 0.828
Pretest APs	One-way ANOVA	Same as above	*F*_(3,107)_ = 0.550	*p* = 0.649

### Persistent LTF was triggered by a second stimulus block delivered at 24 h but not 18 h after initial LTF induction

LTF induced by the standard protocol persists for at least 24 h but substantially decays by 48 h (Zhang et al., 2011; Liu et al., 2017). A second block of this protocol, delivered at 24 h, prolonged LTF to 6 d (Hu et al., 2011). To examine the roles of pCREB1 and pCREB2 in consolidation of LTF, we used a single pulse of 5-HT as a second block, a stimulus that by itself only induces short-term synaptic facilitation (Montarolo et al., 1986; Bartsch et al., 1995, 1998; Zhou et al., 2014). We hypothesized that this stimulus delivered 24 h after a first block of the standard protocol would also prolong LTF, due, at least in part, to a residual trace of elevated pCREB1. However, a second block at 18 h would be expected to yield a different result because, at that time, both pCREB1 and pCREB2 are elevated (Fig. 1*A2*,*B2*). LTF might be enhanced due to CREB1 activation or suppressed if CREB2-mediated repression predominates.

Three groups of SN-motor neuron (MN) cocultures were treated with an initial stimulus block to induce LTF (Fig. 2*A*). At 18 h, one group (*S*_18h_) was given a single 5 min treatment of 5-HT. The second group (*S*_24h_) was treated at 24 h. The third group (*V*_24h_) was treated with 5 min of vehicle to monitor the decay with time of LTF induced by the first block alone. In order to keep the test times consistent across experiments, excitatory postsynaptic potentials (EPSPs) were recorded with 24 h increments, 48 h (2 d posttest), and 72 h (3 d posttest) after the first block.

Similar to previous findings (Hu et al., 2011), *S*_24h_ produced LTF that was enhanced and prolonged compared with LTF produced by the first block alone (*V*_24h_). Surprisingly, a second block at 18 h, *S*_18h_, failed to prolong LTF (Fig. 2*B*,*C*). Two-way repeated-measures (RM) ANOVA with subsequent pairwise comparisons revealed significant differences among the three groups and between the two posttest time points. The increase in EPSP amplitude at 2 d posttest in *S*_24h_ was significantly greater than in *V*_24h_ and *S*_18h_, but no significant difference was found between *V*_24h_ and *S*_18h_. No significant differences were found in basal synaptic strength (pretest) among the three groups; thus the observed differences in facilitation were not due to variable basal strength. These results indicate 24 h, but not at 18 h, is an effective IBI in this system.

### Inhibiting CREB2 activation enabled the second stimulus block at 18 h to prolong LTF

Activation of CREB2 increased at 18 h (Fig. 1*B*), suggesting transcription repression by pCREB2 may prevent the second block delivered at 18 h from effectively enhancing LTF. CREB2 activation is mediated, in part, by p38 MAPK (Guan et al., 2003). Therefore, we hypothesized that inhibition of p38 MAPK would facilitate persistent LTF.

To test this hypothesis, we first confirmed the increase in pCREB2 at 18 h can be blocked by the p38 MAPK inhibitor SB203580 (SB). SB (3 µM) was applied for 1 h to SNs 17 h after the first stimulus block (Fig. 3*A1*). A concentration of 3 µM was used previously to block the effects of p38 MAPK (Liu et al., 2014, 2022; Zhang et al., 2017). Four dishes of SNs were used for each set of experiments: (1) Veh alone (*V*_0h_), (2) standard protocol (*S*_0h_), (3) SB alone, or (4) *S*_0h_ *+* SB. One-way RM ANOVA indicated significant differences in pCREB2 nuclear immunoreactivity among the four groups (Fig. 3*A3*). Pairwise comparisons indicated immunoreactivity to pCREB2 in the *S*_0h_ group was significantly greater than in the *V*_0h_, SB alone, and *S*_0h_ *+* SB groups, indicating SB inhibited 5-HT-induced pCREB2 activation. To examine whether application of SB also affected the activation of CREB1, we repeated the protocol in Figure 3*A* and examined levels of pCREB1. No significant difference was found in levels of pCREB1 between *S*_0h_ and *S*_0h_ + SB groups (Fig. S3*A*), indicating that the SB-mediated decrease in pCREB2 (Fig. 3*A3*) was not associated with a change in pCREB1 levels.

To examine whether suppressing CREB2 activation improves the efficacy of 2-block training with this 18 h IBI, SB was applied 17 h after the first block and remained present through the stimulus at 18 h (Fig. 3*B1*). Four groups of SN-MN cocultures were used: (1) *S*_18h_, to monitor normal LTF induced by two blocks of 5-HT application separated by 18 h; (2) *S*_18h_ + SB, to examine the effect of SB paired with the single 5-HT stimulus at 18 h; (3) *V*_18h_ *+* SB, to examine whether SB alone, applied at 17 h after the first block, but without the second, can enhance/prolong LTF; and (4) NS_0h_ + *S*_18h_ *+* SB, to examine whether, without the first block (NS_0h_), *S*_18h_ paired with SB induces LTF. In Figure 2, the 2 d posttests were performed 30 h after the second block in the *S*_18h_ group, whereas this interval was 24 h in the *S*_24h_ group. Thus, a time-dependent decay in the effectiveness of the second block could potentially contribute to the reduction in EPSP magnitude in the *S*_18h_ group. To address this possibility, EPSPs were measured both 24 and 48 h after the second block (Fig. 3*B1*, Fig. S4).

Remarkably, the *S*_18h_ *+* SB group exhibited substantial LTF 2 d and even 3 d after the initial stimulus block (Figs. 3*B2*,*B3*). Two-way ANOVA with subsequent pairwise comparisons indicated EPSP amplitudes were significantly greater in *S*_18h_ *+* SB, compared with the other three groups. No significant differences in pretest synaptic strength were observed among the groups. Thus, reducing pCREB2 activation at 18 h removes an inhibitory constraint, allowing a second stimulus block to prolong LTF for at least 3 d.

The prolonged LTF observed when CREB2 activation was inhibited during a second stimulus block at 18 h may result, at least in part, from elevated pCREB1 levels. To test this possibility, we measured pCREB1 24 h after a second stimulus block applied at 18 h in the presence of SB and found that pCREB1 levels were significantly increased (Fig. S3*B*).

### A critical temporal window for effective interblock intervals

The initial stimulus block induces a complex pattern of activation of kinases with multiple waves, and these dynamics persist for at least 24 h (Atkins et al., 1998; Muller and Carew, 1998; Liu et al., 2020; Zhang et al., 2023). To gain insights into the ways in which these dynamics influence the effectiveness of a second stimulus block to reinforce LTF, we developed a phenomenological model to capture the essential features of the activation of kinases and transcription factors (Supplemental Information, Figs. S1, S2). The model predicted an effective time window for a second stimulus block, between 20 and 30 h after the first (Fig. S2*D*). To test whether this window does close after 30 h, we repeated the experiment of Figure 2 but with the second stimulus block delivered 32 h after the first (*S*_32h_). A 24 h IBI induced LTF persisting to 2 d posttest (Fig. 4*A*). However, the 32 h IBI failed to induce persistent LTF. Two-way RM ANOVA with subsequent pairwise comparisons revealed significant differences between the two groups and between the two posttest times.

### CREB1 and CREB2 dynamics and the predicted critical temporal window

The model predicted that the closure of the effective time window for a second stimulus block at ∼30 h after the initial stimulus was due to the deactivation of transcription factors. To empirically test this prediction, changes in pCREB1 (Fig. 4*B2*,*B3*) and pCREB2 (Fig. 4*C3*) at 18, 24, and 32 h after the first stimulus block were compared. pCREB1 increased at 18 h, declined at 24 h, and returned to baseline at 32 h. pCREB2 also increased at 18 h, dropped to basal at 24 h, but, surprisingly, increased at 32 h. One-way ANOVA for pCREB1 revealed a significant overall difference among the three time points. Subsequent pairwise comparisons revealed that pCREB1 levels at 18 h were significantly different from 32 h, but not from 24 h. Similarly, pCREB1 levels at 32 h were not significantly different from 24 h. One-way ANOVA for pCREB2 also revealed a significant overall difference among all three time points. Subsequent pairwise comparisons revealed that pCREB2 levels at 18 and 32 h differed significantly from 24 h, but not from each other.

### A similar critical temporal window for LTEE

Sensitization training and 5-HT also elicit LTEE of SNs (Dale et al., 1987; Cleary et al., 1998; Liu et al., 2011a), another correlate of LTM (Zhang and Linden, 2003; Mozzachiodi and Byrne, 2010). The standard 5-HT protocol produces LTEE in isolated sensory neurons that persists for at least 24 h, but decays by 48 h post-treatment (Liu et al., 2011a). To test whether LTEE could be enhanced and prolonged by a second stimulus block, and whether the timing of this second block follows a similar temporal window as observed for LTF, three groups of SNs were examined: (1) *S*_18h_, (2) *S*_24h_, and (3) *S*_32h_ (Fig. 5*A*). Excitability was assessed by counting the number of action potentials elicited by a 2 s duration, 0.5 nA depolarizing current pulse, measured before the initial stimulus block (pretest), 2 and 3 d posttest. LTEE was greater after a second stimulus block delivered at 24 h, than after a second block at 18 or 32 h (Fig. 5*B*,*C*). Two-way RM ANOVA with subsequent pairwise comparisons revealed significant differences among these three groups. Post hoc tests indicated LTEE was greater in the *S*_24h_ group than in *S*_18h_ and *S*_32h_, with no significant difference between *S*_18h_ and *S*_32h_. No significant differences in basal excitability (pretest) were found among groups. Therefore, LTEE exhibits a similar critical temporal window as LTF.

The failure of a second block at 18 or 32 h to enhance LTF and LTEE could be due to a diurnal modulation that affects the ability of 5-HT to activate second-messenger cascades in the cultured SNs. To examine this possibility, we compared the ERK activation produced by a single pulse of 5-HT, delivered at 8:00 A.M., 2:00 P.M., and 10:00 P.M., without a preceding stimulus block (Fig. S5). These time points correspond to 18, 24, and 32 h after the initial 5-HT block in the electrophysiological experiments, the same times at which the single 5-HT pulse was delivered. Previous studies found that phosphorylated ERK (pERK) was elevated 45 min after a single stimulus (Philips et al., 2007, 2013b; Kopec et al., 2015; Zhang et al., 2017). Consistently, we observed increased pERK 45 min after 5-HT, for all three time points (Fig. S5*B*1). One-way ANOVA revealed no significant differences among the three time points (Fig. S5*B*2).

## Discussion

### Dynamic competition between CREB1 and CREB2 supports a critical temporal window for effectiveness of a second stimulus block

A second stimulus block delivered 24 h after the first significantly enhanced LTF at 2 d posttest (Fig. 2*B*,*C*, *S*_24h_ group), consistent with previous studies that multiple blocks of training or of 5-HT treatment, spaced 24 h apart, produce longer-lasting LTM and LTF in *Aplysia*, compared with a single block. We also expected that a second stimulus block at 24 h would enhance LTEE, based on the similarity in induction signaling pathways for LTF and LTEE (Liu et al., 2011a), and this enhancement was observed. However, IBIs of 18 h (Fig. 2*B*,*C*, *S*_18h_ group) or 32 h (*S*_32h_ group) did not enhance or prolong LTF or LTEE. These 2 d posttests were performed at 30 h after the second block in the *S*_18h_ group, but at 24 h in the *S*_24h_ group. Thus, time-dependent decay in the effectiveness of the second block could potentially reduce the magnitude of the EPSP observed in *S*_18h_ group. However, when comparing EPSPs from this *S*_18h_ group with the combined *S*_24h_ data from Figures 2*C* and 4*A3*, the 24 h IBI was still more effective than 18 h (Fig. S4), suggesting that the failure of the 2-block protocol with an 18 h IBI to enhance LTF is not associated with time-dependent decay of the second stimulus block. In addition, in the *S*_32h_ group in Figure 4*A*, the 2 d posttest was given just 16 h after the second block. If a decaying process initiated by the second block was responsible for the reduced LTF, the EPSP at 2 d posttest would be expected to be greater or equal to that produced by a second block at 24 h, contrary to the results.

Our data suggest these spacing effects are due, at least in part, to the dynamics of competition between the transcription activator CREB1 and repressor CREB2 (Fig. 4). A residual trace of pCREB1 24 h after the first stimulus block (Fig. 1*A*3,1*C*) may facilitate the effect of the second. The failure of a 5-HT pulse to prolong LTF when delivered at 18 h or 32 h suggests that, at these times, repression of transcription by CREB2 predominates.

Consistently, inhibiting p38 MAPK at 18 h, to block CREB2 activation, allowed a second block to prolong LTF for at least 3 d (Fig. 3*B*), an effect even greater than that observed with the 2-block protocol with 24 h IBI (Fig. 2). Notably, although SB blocked the increase in pCREB2 at 18 h (Fig. 3*A3*), inhibition of CREB2 activation between 17 and 18 h did not significantly alter pCREB1 levels (Fig. S3*A*). However, when SB was combined with a second stimulus block delivered at 18 h, pCREB1 was significantly increased 24 h later (Fig. S3*B*), suggesting that elevated pCREB1 at 24 h contributes to the persistent LTF observed in the *S*_18h_ *+* SB group at 2 and 3 d posttests.

Together, these results suggest that, although the LTF-promoting kinase cascades (PKA, ERK, and RSK) are activated at 18 h, the inhibitory effect of p38 MAPK dominates. Dominance of p38 MAPK was also observed previously, when SNs received simultaneous inputs of the facilitating transmitter 5-HT and the inhibitory transmitter FMRFamide (Phe-Met-Arg-Phe-NH2). Long-term synaptic depression resulted due to activation of CREB2 by FMRFamide via p38 MAPK (Guan et al., 2002, 2003).

To gain insights into the ways in which these dynamics influence the effectiveness of a second stimulus block to reinforce LTF, we developed a phenomenological model to capture the essential features of the activation of kinases and transcription factors (Figs. S1, S2). The model predicted an effective time window for a second stimulus block, between 20 and 30 h after the first (Fig. S2*D*). As described in Supplemental Information, this model incorporates complex interactions, and feedback loops, that regulate the activities of kinases and transcription factors necessary for LTF. Simulations captured the salient features of the dynamics of activation of kinases and transcription factors after the first stimulus block (Figs. S1, S2). The simulation results suggest that the efficacy of the second stimulus block cannot be ascribed to one factor but is determined by competing effects of positive feedback loops, involving transcription activators, and negative feedback loops, involving transcription repressors. The model predicted that a second stimulus block delivered at 32 h would fail to induce persistent LTF because positive feedback, mediated by activation of CREB1, is no longer operative at this time, as pCREB1 has returned to basal levels. These predictions were empirically verified (Fig. 4*A*,*B*). The model also predicted that negative feedback, mediated by pCREB2-dependent repression, was no longer operative at 32 h. However, this prediction was not supported by the empirical data, as pCREB2 was elevated at this time (Fig. 4*C3*).

The surprising late (32 h) activation of CREB2 suggests that the closure of the eligibility window is not simply due to the fading of a CREB1 trace but also to an increase in repression of transcription. One possibility is the engagement of protein kinase C (PKC). Hu et al. (2011) found that activation of PKC and regulation of protein synthesis were both required for persistent LTF induced by a second block at 24 h, although they did not explore the effect of shorter or longer IBIs. Further empirical and computational analyses of the dynamics of kinases and transcription factors during this later time domain, and of signaling pathways triggered by the second block, are necessary.

The timing of the second stimulus block is also important for enhancing LTEE, and the windows of eligibility for LTEE and LTF enhancement were remarkably similar. The critical window for LTEE was observed in isolated SNs, indicating the molecular timer mechanism is intrinsic to individual neurons. For LTEE, and likely for LTF, the postsynaptic motor neuron is not required. Additional studies are needed to examine the possible roles of CREB2, p38 MAPK, and PKC in determining the temporal window for LTEE enhancement.

LTM in *Aplysia* is affected by a diurnal cycle with better learning during the day than at night (Fernandez et al., 2003; Lyons et al., 2005), raising the possibility that the window of eligibility for the multiblock protocols could be a consequence of some diurnal modulation of the cultured neurons. However, our data did not support this hypothesis. First, cultures during the incubating period were kept in an incubator with minimal exposure to the natural light/dark cycle. Second, pERK levels in SNs were consistently elevated by 5-HT regardless of whether it was delivered in the morning, afternoon, or night (Fig. S3), with no significant difference between these time points. Third, coapplication of a p38 MAPK inhibitor with the second stimulus block at 18 h effectively prolonged LTF, indicating that the kinase cascades are activated at this time.

### Implications for enhancement of learning over multiple days

Our data suggest an initial stimulus activates a cellular timer, based in part on CREB1 and CREB2 dynamics, that constrains enhancement of initial learning for at least 18 h and rather suddenly releases the constraint near 24 h poststimulus. But soon after 24 h, a trace based in part on prolonged CREB1 activity fades, while inhibition from CREB2 reemerges, counteracting the effectiveness of a second stimulus block at later times (e.g., 32 h).

In virtually every case of multiday training trials, the interval is set at 24 h (Table 1). Our studies, combining model predictions and empirical tests, suggest the selection of 24 h may be fortuitous and that shorter or longer intervals might be less effective. Timers and clocks constructed from biochemical components are ubiquitous in cell biology (Gliech and Holland, 2020), but to our knowledge, an intrinsic molecular timer has not previously been shown to regulate the effectiveness of multiday learning trials or a window of eligibility for those trials.

It will be interesting to examine the extent to which cellular timers initiated by learning exist in other nervous systems, and their ecological significance. Molecular mechanisms of memory, including the roles of the CREB family of transcription factors, are substantially conserved between invertebrates and vertebrates (Bourtchouladze et al., 1998; Taubenfeld et al., 2001; Barco et al., 2006; Porte et al., 2008; Alberini, 2009; Chen et al., 2012; Philips et al., 2013a). Thus, the efficacy of repeated learning experiences may be controlled by a 24 h biological “hour-glass” timer triggered by the initial experience, which sets the “right time” to reinforce learning. Such a timing mechanism may yield better learning, if training or repeated experiences recur at the same time of each day.
